# Simultaneous differential detection of H5, H7 and H9 subtypes of avian influenza viruses by a triplex fluorescence loop-mediated isothermal amplification assay

**DOI:** 10.3389/fvets.2024.1419312

**Published:** 2024-07-02

**Authors:** Qing Fan, Zhixun Xie, Junke Zhao, Jun Hua, You Wei, Xiaofeng Li, Dan Li, Sisi Luo, Meng Li, Liji Xie, Yanfang Zhang, Minxiu Zhang, Sheng Wang, Hongyu Ren, Lijun Wan

**Affiliations:** Guangxi Key Laboratory of Veterinary Biotechnology, Key Laboratory of China (Guangxi)-ASEAN Cross-Border Animal Disease Prevention and Control, Ministry of Agriculture and Rural Affairs of China, Guangxi Veterinary Research Institute, Nanning, China

**Keywords:** AIV, H5, H7, H9, TLAMP, probe, differential diagnosis

## Abstract

H5, H7, and H9 are pivotal avian influenza virus (AIV) subtypes that cause substantial economic losses and pose potential threats to public health worldwide. In this study, a novel triplex fluorescence reverse transcription-loop-mediated isothermal amplification (TLAMP) assay was developed in which traditional LAMP techniques were combined with probes for detection. Through this innovative approach, H5, H7, and H9 subtypes of AIV can be simultaneously identified and differentiated, thereby offering crucial technical support for prevention and control efforts. Three primer sets and composite probes were designed based on conserved regions of the haemagglutinin gene for each subtype. The probes were labelled with distinct fluorophores at their 3′ ends, which were detached to release the fluorescence signal during the amplification process. The detection results were interpreted based on the colour of the TLAMP products. Then, the reaction conditions were optimized, and three primer sets and probes were combined in the same reaction system, resulting in a TLAMP detection assay for the differential diagnosis of AIV subtypes. Sensitivity testing with *in vitro*-transcribed RNA revealed that the detection limit of the TLAMP assay was 205 copies per reaction for H5, 360 copies for H7, and 545 copies for H9. The TLAMP assay demonstrated excellent specificity, no cross-reactivity with related avian viruses, and 100% consistency with a previously published quantitative polymerase chain reaction (qPCR) assay. Therefore, due to its simplicity, rapidity, sensitivity, and specificity, this TLAMP assay is suitable for epidemiological investigations and is a valuable tool for detecting and distinguishing H5, H7, and H9 subtypes of AIV in clinical samples.

## Introduction

Avian influenza (AI) is a zoonotic disease caused by the avian influenza virus (AIV) that results in human infections and economic losses each year (1). AIV is a type A influenza virus in the family *Orthomyxoviridae* with a negative-sense segmented RNA genome. Based on differences in the haemagglutinin (HA) and neuraminidase (NA) antigens, 18 HA (H1–H18) and 11 NA (N1–N11) subtypes have been identified (2, 3). These strains are classified based on their pathogenicity as either a high-pathogenicity AIV (HPAIV) or a low-pathogenicity AIV (LPAIV). Strains H5 and H7 (particularly H5N1 and H7N9 subtypes) are HPAIVs that can cause severe illness and mortality in domestic poultry and humans (4, 5). In 1997, the H5N1 virus infected 18 people in Hong Kong, resulting in 6 fatalities (6). The emergence of the H7N9 avian influenza viruses in China in early 2013 caused five waves of human infection from 2013–2017, with a total of 1,568 cases and 615 fatalities (7). LPAIVs typically show mild or no symptoms in birds but have the potential to cause pathogenic avian influenza through antigenic drift or shift and pose the risk of human infection (8). The H9 subtype of AIV, which has been classified as a LPAIV, is reportedly capable of infecting humans (9). This strain contributes internal genes to H5 and H7 viruses with human infectivity, such as H7N9 and H5N6, making it an important candidate that could cause new influenza pandemics in humans (10–12). The H5, H7, and H9 subtypes of AIV are crucial in poultry (13–15), emphasizing the urgent need to develop quick and sensitive diagnostic tools for the early detection of AIVs, especially the H5, H7, and H9 subtypes.

Molecular biology-based diagnostic methods using polymerase chain reaction (PCR) technology, such as reverse transcription PCR (RT-PCR), quantitative PCR (qPCR), and GeXP, have become commonplace for AIV detection and genotyping (16–19). However, these methods are expensive and rely on sophisticated laboratory instruments, including real-time fluorescence PCR and GeXP instruments; therefore, these methods are impractical for use in rural areas. To prevent and control infectious diseases, swift and accurate diagnostics must be performed immediately in endemic areas, which underscores the need for timely point-of-care (POC) testing platforms to meet the evolving challenges of disease detection. Amongst numerous nucleic acid amplification assays, loop-mediated isothermal amplification (LAMP) stands out in terms of its sample-to-answer time, sensitivity, specificity, cost, robustness, and accessibility, making it ideal for field-deployable diagnostics in resource-limited regions (20). One of the greatest opportunities for LAMP assays as a POC tool is their ability to eliminate nucleic acid purification or extraction steps and perform direct amplification from pretreated crude samples. Reverse-transcription-LAMP (RT-LAMP) sample pretreatment methods, such as thermal lysis, proteinase K and RNAsecure, can inactivate or inhibit RNase in the sample and lyse the virus particles (21). Another advantage of using the LAMP assay as a POC test is the ability to visually detect amplicons. Techniques with a visual endpoint can allow the direct detection of amplicons using probes for a downstream immunoassay or allow the measurement of amplification indirectly by detecting the formation of pyrophosphate precipitates (22, 23).

The sensitivity and simplicity of LAMP methods generally fall between those of qPCR and rapid antigen tests. At present, LAMP technology is widely used to diagnose a variety of diseases. RT-LAMP assays that can detect SARS-CoV-2, the virus that causes COVID-19, have already penetrated commercial markets and were authorized by the US Food and Drug Administration (FDA) for emergency clinical POC diagnosis (21). Moreover, these assays have found applications in three settings: highly complex laboratories, POC testing, and at home. However, all commercially available LAMP kits or settings can detect only a single target and fail to achieve the differential diagnosis of multiple targets. In this study, the conventional LAMP assay was innovatively improved by incorporating a quencher-fluorophore composite probe labelled with different fluorophores that display distinct colours at the corresponding wavelengths. Such modification led to the establishment of a groundbreaking triplex fluorescence LAMP (TLAMP) assay capable of simultaneously identifying H5, H7, and H9 subtypes in a single tube. The TLAMP results are directly interpretable by the naked eye and more accurate and intuitive than those of traditional methods. This study offers technical support for advancing AIV POC diagnostic devices as well as innovative strategies for utilizing LAMP technology to achieve multitarget detection.

## Materials and methods

### Ethics statement

This study was approved by the Institutional Animal Care and Use Committee (IACUC) of Guangxi Veterinary Research Institution (GVRI). Sample collection was conducted based on protocol #2023C004 issued by the IACUC of GVRI. All samples were collected from live chickens on approved farms by well-trained veterinarians. All methods were performed in accordance with the relevant guidelines and regulations.

### Strain sources and nucleic acid extraction

The viruses used in this study are outlined in Table 1. RNA was extracted from four specific pathogen-free (SPF) chicken swab samples and served as the negative control. Nucleic acids from the entities listed in Table 1 were extracted with the *EasyPure* Viral DNA/RNA Kit (TransGen Biotech, Beijing, China). The DNA and cDNA were stored at −30°C, and the RNA was stored at −70°C until use. Two microlitres of DNA/cDNA/RNA was used as a template to evaluate the specificity of the TLAMP assay.

**Table 1:** Viruses used and the corresponding TLAMP results.

Strain/sample type	Source	TLAMP results		
		H5	H7	H9
A/turkey/GA/209092/02 (H5N2) AIV cDNA	UC, USA	+	−	−
A/chicken/QT35/98 (H5N9) AIV cDNA	UP, USA	+	−	−
A/chicken/NY/273874/03 (H7N2) AIV cDNA	UC, USA	−	+	−
A/duck/HK/47/76 (H7N2) AIV cDNA	UHK, China	−	+	−
A/duck/42848/07 (H7N7) AIV cDNA	UP, USA	−	+	−
A/dove/Guangxi/408P55/2020 (H9N2) AIV	GVRI, China	−	−	+
A/chicken/Guangxi/449C11/2021 (H9N2) AIV	GVRI, China	−	−	+
A/duck/Guangxi/413D40/2020 (H9N2) AIV	GVRI, China	−	−	+
A/duck/Guangxi/291D16/2017 (H1N6) AIV	GVRI, China	−	−	−
A/broiler/PA/117/04 (H2N2) AIV	UP, USA	−	−	−
A/goose/Guangxi/318G39/2018 (H3N2) AIV	GVRI, China	−	−	−
A/duck/Guangxi/201D19/2016 (H4N8) AIV	GVRI, China	−	−	−
A/duck/Guangxi/330D18/2018 (H6N6) AIV	GVRI, China	−	−	−
A/turkey/ont/6118/68 (H8N4) AIV	UHK, China	−	−	−
A/duck/HK/876/80 (H10N3) AIV	UHK, China	−	−	−
A/duck/HK/661/79 (H11N3) AIV	UHK, China	−	−	−
A/duck/HK/862/80 (H12N5) AIV	UHK, China	−	−	−
A/gull/Md/704/77 (H13N5) AIV	UHK, China	−	−	−
A/mallard duck/Astrakhan/263/82 (H14N5) AIV	UC, USA	−	−	−
A/shearwater/Australia/2576/79 (H15N9) AIV	UC, USA	−	−	−
A/shorebird/Delaware/168/06 (H16N3) AIV	UC, USA	−	−	−
Newcastle disease virus (NDV), F48	CIVDC, China	−	−	−
Infectious bronchitis virus (IBV), M 41	CIVDC, China	−	−	−
Avian reovirus (ARV), S1133	CIVDC, China	−	−	−
Avian infectious laryngotracheitis virus (ILTV), Beijing	CIVDC, China	−	−	−
Chicken infectious anaemia virus (CIAV), GX1804	GVRI, China	−	−	−
Marek’s disease virus (MDV) live-attenuated vaccine, CVI988	Boehringer Ingelheim, China	−	−	−
Fowl aviadenovirus serotype 4 (FAdV-4), GX005	GVRI, China	−	−	
Chicken parvovirus (ChPV), GX-ChPV-1, DNA	GVRI, China	−	−	−
Negative control, SPF chickens	Boehringer Ingelheim, China	−	−	−

GVRI, Guangxi Veterinary Research Institute; UHK, University of Hong Kong; UP, University of Pennsylvania; UC, University of Connecticut; CIVDC, China Institute of Veterinary Drug Control; CIVDC, China Institute of Veterinary Drug Control.

### Primer and probe design and preparation of the composite probe (FIP-FD)

Thousands of AIV H5, H7, and H9 sequences were procured from the influenza virus database housed at the National Center for Biotechnology Information (NCBI).^1^ Using MegAlign within DNASTAR-Lasergene 8.0 software, the conserved regions and sequences of HA subtypes in AIVs were meticulously identified. Subsequently, Primer Premier 5 and PrimerExplorer V5 online software^2^ were used to design three sets of TLAMP primers and the corresponding FD probes, with each set specifically tailored to target the identified subtype.

The inner primer contained two primers in series, FIP (F1c + F2) and BIP (B1c + B2). The probe FD is a complementary sequence to the F1c fragment of FIP. The 3′ end of the FD probe of each subtype was labelled with a fluorescent group, each with a different emission wavelength (FAM, Cy5, or Cy3), while the 5′ end of the inner primer FIP was labelled with the corresponding quenching group from the BHQ series. Before the reaction, FIP-quenched (FIP-Q) and FD-fluorescence (FD-F) were annealed, forming a “fluorescence-quenched” composite probe (hereafter referred to as FIP-FD) that did not fluoresce prior to the reaction. Throughout the TLAMP assay, FIP maintained its role as an internal primer to guide amplification. However, during synthesis from the reverse direction directed by BIP, the FD probe detached from FIP-FD, releasing a discernible fluorescence signal (Supplementary Figure S1). After the reaction, the detection results were interpreted based on the colour of the TLAMP product, highlighting the innovative nature of TLAMP.

Before the reaction, the fluorescence-quenched annealed composite probes were prepared by mixing 50 μmol/L FIP-Q and 50 μmol/L FD-F and heating at 90°C for 5 min, after which the mixture was gradually cooled to room temperature to yield the annealed FIP-FD composite probe, which was stored at −20°C until use. The primers and probes used were synthesized by Takara Biomedical Technology (Dalian, China) Co., Ltd., and the sequences are provided in Table 2.

**Table 2:** Sequences of the primers and probes used in the TLAMP assay.

Primer-probe	Sequence (5′–3′)	TM (°C)
H5-F3	TTCCATGACTCAAATGTCAAG	57.6
H5-B3	GCTAGGGAACCCGCCACT	59.7
H5-FIP-Q	BHQ1-CATACATTCATTATCACATTTGTGGACTACAGCTTAGGGA**Y**AATGCAAA^b^	63.2/59.6^a^
H5-BIP	GAAAGTGTGAGAAATGGGACGTTCTTCCCTTTTTA**R**TCTTGCTT^b^	60.5/55.7^a^
H5-Floop	CGAAACAACCATTACCCAGCTC	61.3
H5-Bloop	ATGACTACCCCCAGTATTCAGAAG	58.7
H5-FD	ATGTGATAATGAATGTATG-FAM	39.2
H7-F3	TCACATACAATGGAATAAGAAC	55.2
H7-B3	CCCATATAGCTTGGTTTGCT	57.2
H7-FIP-Q	BHQ3-CTGTGTTTGACAGGAGCCATTTCGTGACCAGTGCATGTAGG	56.5/61.9^a^
H7-BIP	TTCCCGCAGATGACTAA**R**TCATAAGTTGAAACGGAATG**R**TGGA^b^	56.7/61.3^a^
H7-Floop	TCTGCATAGAATGAAGATCCTGATC	59.7
H7-Bloop	CCAGCTATAGTAGTATGGGG	57.8
H7-FD	CTCCTGTCAAACACAG-Cy5	41.5
H9-F3	ACAAAATGAACAAGCAGTATGAAAT	57.6
H9-B3	TCCATGCATTGGTCATCACATT	60.8
H9-FIP-Q	BHQ2-GATCGTCAATCTTATTGTTAATCATGATCATGAATTCAGCGAGG	55.0/55.0^a^
H9-BIP	TATGGGCATATAATGCAGA**R**TTGCTTTTGCATCATGCTCATCGA^b^	56.8/60.9^a^
H9-Floop	TTGTTAATCATGTTAAGTCTAGTTT	57.1
H9-Bloop	AGAATTGCTAGTTCTGCTTGAAAA	58.0
H9-FD	AACAATAAGATTGACGATC-Cy3	44.0

a The inner primer consists of two primers joined together, such as FIP=F1c + F2 and BIP=B1c + B2, so there are two annealing temperatures.

b The bold font indicates degenerate sites (R: A/G; Y: C/T).

### Preparation of the standards

The HA genes of H5, H7 and H9 were individually cloned and inserted into the *pEASY* T-18T vector (TransGen Biotech, Beijing, China) to generate three recombinant plasmids, each of which was verified via sequencing. Subsequently, the recombinant plasmid that contained the target gene was isolated using the *EasyPure*^®^ HiPure Plasmid MiniPrep Kit (TransGen Biotech, Beijing, China) and transcribed into purified RNA using the *In vitro* Transcription T7 Kit (Takara, Dalian, China). The concentrations of the obtained purified RNA transcripts were determined using a NanoDrop 2000 (Thermo Fisher Scientific, Waltham, MA, United States). The number of RNA transcripts was calculated following a previously described formula (17). Equal amounts of the three RNA transcripts were then mixed and serially diluted twofold with TE buffer to prepare the RNA standards, the concentrations of which are listed in Table 3. This twofold series of diluted RNA standards comprising eight concentrations of RNA was used to evaluate the sensitivity of the TLAMP assay.

**Table 3:** The TLAMP assay was repeated 10 times for each standard.

RNA standard	H5 RNA (copies/μL)	No. of H5-positive samples	H7 RNA (copies/μL)	No. of H7-positive samples	H9 RNA (copies/μL)	No. of H9-positive samples	No. of tests
1	1,600	10	2,820	10	4,130	10	10
2	800	10	1,410	10	2,065	10	10
3	400	10	705	10	1,033	10	10
4	200	10	353	10	516	10	10
5	100	9	176	10	258	9	10
6	50	6	88	2	129	5	10
7	25	0	44	0	65	0	10
8	12.5	0	22	0	32	0	10

### Clinical sample collection

From January to October 2023, 240 broilers, including Ma chicken, Sanhuang chicken, and Fufeng native chicken breeds, were randomly selected from live bird markets (LBMs) in Guangxi, China. The sampled broilers exhibited good health without any signs of disease, and swab samples from the cloacae and larynges were collected. The swab specimens were directly eluted with 1 mL of PBS, and RNA was extracted from the centrifuged supernatant using the MagicPure^®^ Simple Viral DNA/RNA Kit (TransGen Biotech, Beijing, China). cDNA from different tissue samples of eight H5N2-infected SPF chickens was donated by the University of Connecticut, and cDNA from different tissue samples of three H7N9-infected SPF chickens was donated by China Agricultural University (19). The swab and tissue samples were evaluated by performing the TLAMP assay and previously published H5, H7, and H9 qPCR assays (16). qPCR-positive products were subsequently forwarded to BGI Company (Shenzhen, China) for sequencing to rule out false-positive results. A comparative analysis of the TLAMP results, qPCR results, and sequencing data was conducted to assess the clinical applicability of the TLAMP method.

## Results

### TLAMP reaction system

The TLAMP reaction mixture was established with a volume of 25 μL after each reaction condition was optimized. The optimal ratio of outer primer to inner primer to loop primer for the H5 and H7 subtypes was determined to be 1:8:2, while for H9, this ratio was 1:16:4. The concentrations of each component are presented in Table 4, which highlights the adjustments made to increase the efficacy of the TLAMP reaction system. Negative and positive controls were also used for each reaction.

**Table 4:** TLAMP reaction system.

Reagent	Concentration in the 25 μL system (μmol/L)		
WarmStart Multi-Purpose LAMP/RT-LAMP 2× Premix	12.5 μL		
Bst 2.0 WarmStart DNA polymerase	2 U		
Subtype	H5	H7	H9
FIP-FD	0.133	0.067	0.067
Unlabelled FIP	—	0.1	0.267
FIP-Q	0.134	0.1	0.2
BIP	0.266	0.266	0.534
F3	0.033	0.033	0.033
B3	0.033	0.033	0.033
Floop	0.066	0.066	0.132
Bloop	0.066	0.066	0.132
ddH_2_O	Add to a total volume of 25 μL		

The TLAMP reaction was performed with amplification at 63°C for 75 min followed by termination at 80°C for 5 min. A real-time turbidimeter (LA-320; Eiken Chemical Co., Ltd., Tokyo, Japan) or water bath was used.

To interpret the results, an image analyser (Universal Hood III, 731BR01622, Bio-Rad, Hercules, CA, United States) was used after the reaction. The reaction products were compared with the negative control. The results were determined based on the colour of the fluorophore in the reaction tube at the corresponding channels. The positive results were determined as follows: green fluorescence in the 520 nm channel indicated H5-positive samples, red fluorescence in the 670 nm channel indicated H7-positive samples, and blue fluorescence in the 570 nm channel indicated H9-positive samples; overlapping fluorescence in multiple channels was considered indicative of coinfected samples. Notably, in the visible spectrum, Cy3 appears orange, and to distinguish Cy3 from the overlapping colours of FAM (green) and Cy5 (red), Cy3 is shown in blue in the output image (Supplementary Figure S2).

### Identifying the optimal proportions of the components in the composite probe FIP-FD among the total FIP primers

**Figure 1:**
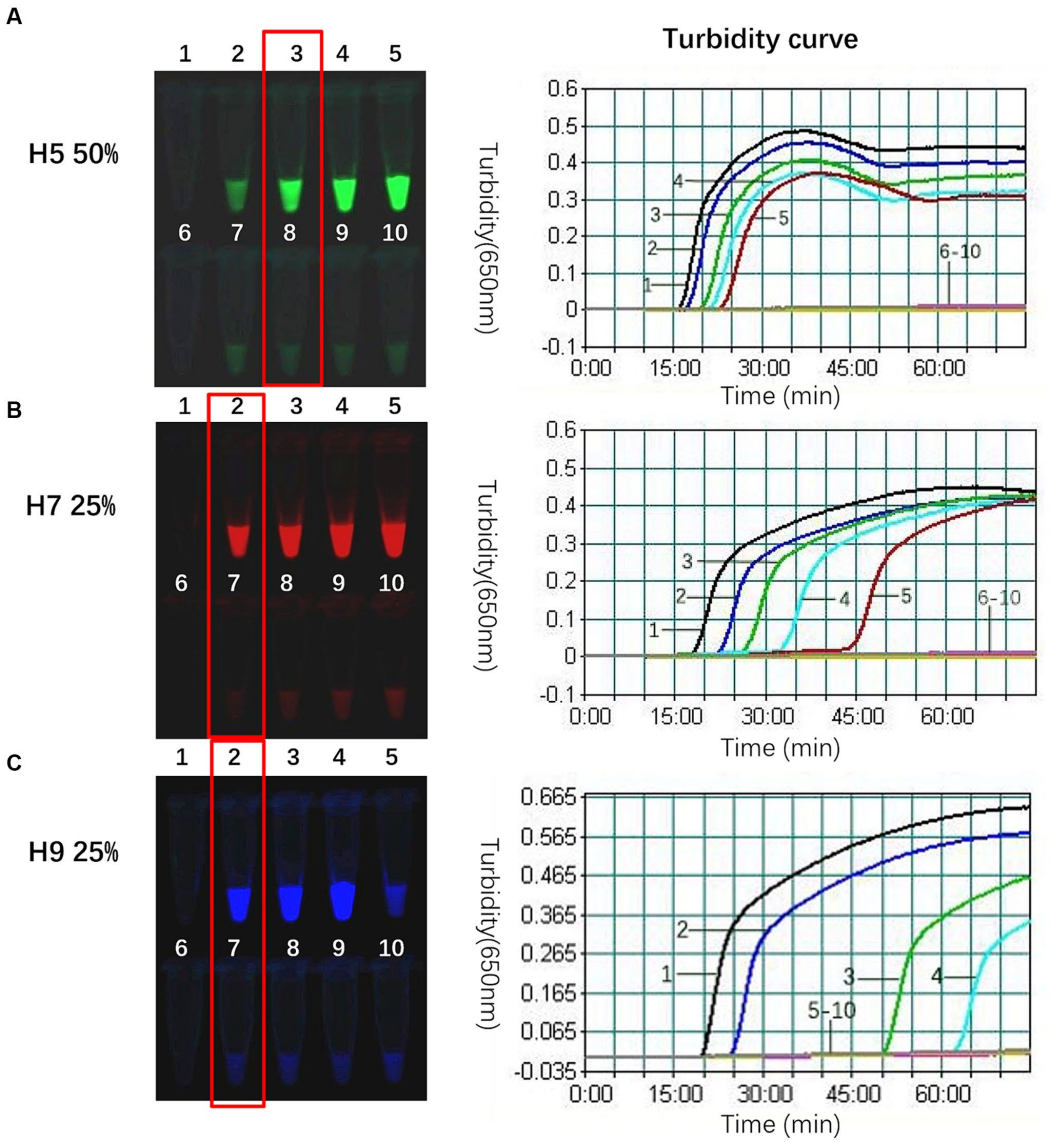
Optimal proportion of FIP-FD among the total FIP primers for each subtype. **(A)** The optimal proportion of H5-FIP-FD among the total H5-FIP primers in the 520 nm channel was 50% at a H5-FIP-FD concentration of 0.133 μmol/L and an unlabelled H5-FIP concentration of 0.134 μmol/L and an initiation time of 20 min. **(B)** The optimal proportion of H7 in the 670 nm channel was 25% with 0.067 μmol/L H7-FIP-FD, 0.2 μmol/L unlabelled H7-FIP, and an initiation time of 23 min. **(C)** The optimal proportion of H9 in the 570 nm channel was 25% with 0.067 μmol/L H9-FIP-FD, 0.2 μmol/L unlabelled H9-FIP, and an initiation time of 25 min. When the H9 proportion was 100%, only 0.267 μmol/L H9-FIP-FD was used with no H9-FIP, and the reaction was completely suppressed. 1: FD-FIP composite probe among the total FIP = 0; 2: 25%; 3: 50%; 4: 75%; and 5: 100%; 6, 7, 8, 9, and 10 are the negative controls for the corresponding proportions.

The composite probe FIP-FD was created by annealing the inner primer FIP with the probe FD. The ability of FIP-FD to guide new chain synthesis was attenuated by the fluorophore and quenching group, and the use of FIP-FD alone in the reaction system inhibited amplification. Previous studies have indicated that FIP-FD must be combined with a specific proportion of the unlabelled quenched group FIP (hereinafter referred to as unlabelled FIP) to effectively mitigate this inhibition (24–26). In the single-template system, the proportions of FIP-FD among the total FIP primer were set to 0, 25, 50, 75, and 100%, as outlined in Table 5. A real-time turbidimeter was used to generate a turbidity curve, with the *x*-axis denoting the reaction time and the *y*-axis indicating the turbidity intensity, and the curve reflected the quantity of the white precipitate byproduct from the TLAMP assay, magnesium pyrophosphate. The parameters that gave the most significant negative to positive contrast in fluorescence after the reaction and the shortest initiation time were determined to be the optimal reaction conditions. Specifically, the optimal proportion of the H5 subtype was 50%, while the optimal proportion of both the H7 and H9 subtypes was 25%. The results of this optimization process are illustrated in Figure 1.

**Table 5:** Proportions of the FIP-FD composite probes among the total FIP primers.

Proportion	0		25%		50%		75%		100%	
FIP-FD (μmol/L)	0		0.067		0.133		0.200		0.267	
Unlabelled FIP (μmol/L)	0.267		0.200		0.134		0.067		0	
Sample No. in Figure 1	+	−	+	−	+	−	+	−	+	−
	1	6	2	7	3	8	4	9	5	10

### Determination of the optimal working concentration of FIP-Q and unlabelled FIP for each subtype

**Figure 2:**
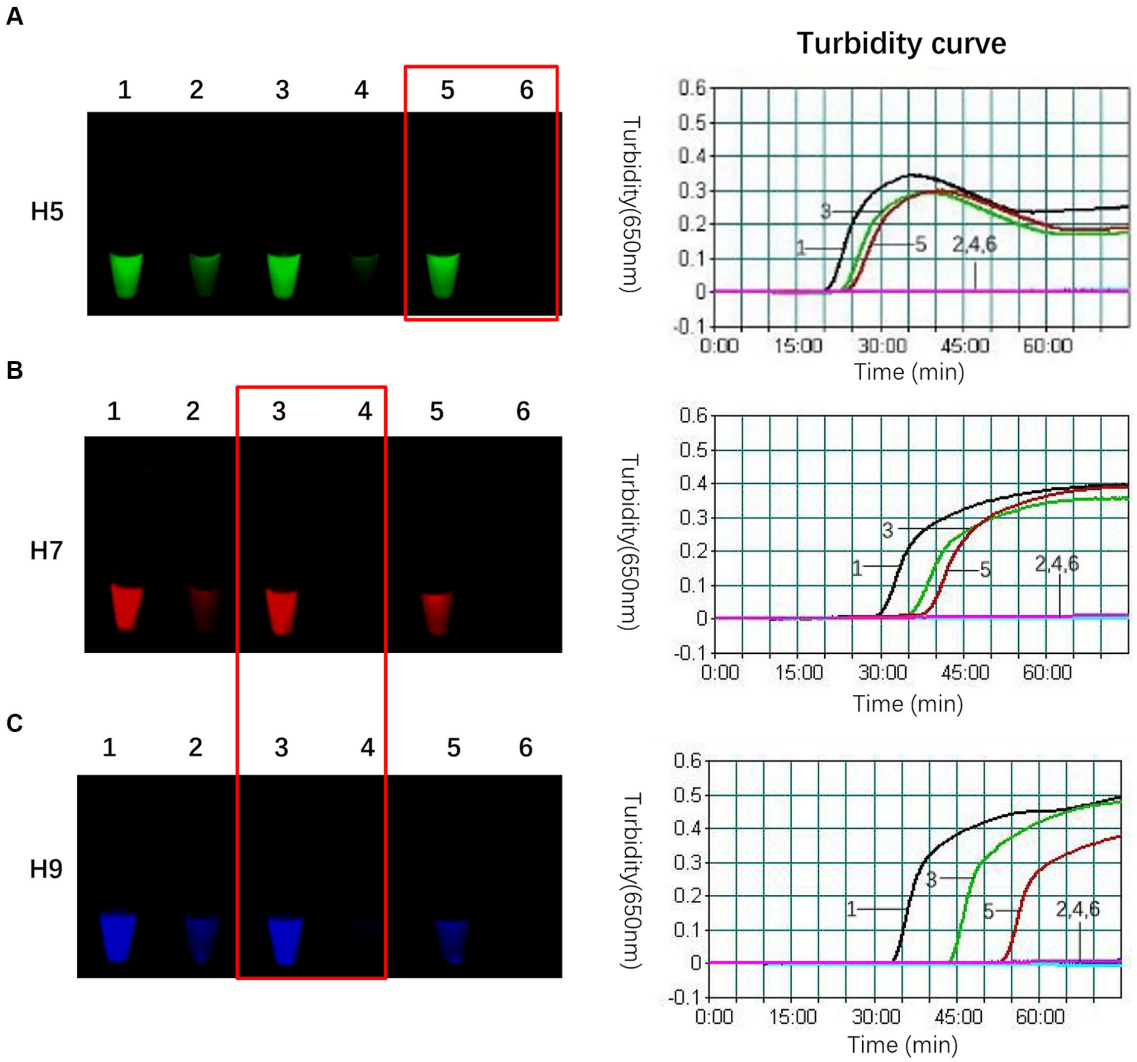
Optimal concentrations of FIP-Q and unlabelled FIP for each subtype. **(A)** When only 0.134 μmol/L H5-FIP-Q was used instead of unlabelled H5-FIP in the H5 primer set, the reaction initiation time was 24 min, the background fluorescence was the lowest, and the difference between the negative and positive samples was the most pronounced. **(B)** In the optimal reaction, the concentrations of the H7 primer group were 0.1 μmol/L unlabelled H7-FIP and 0.1 μmol/L H7-FIP-Q, the initiation time was 35 min, and the difference between the negative and positive samples was the most pronounced. **(C)** In the optima reaction, the concentration of the H9 primer set was the same as that of the H7 primer set with a 43 min initiation time, and the difference between the negative and positive samples was the most pronounced. 1: Unlabelled FIP only (no FIP-Q was present); 2: the negative control of 1; 3: FIP-Q: unlabelled FIP = 1:1; 4: the negative control of 3; 5: FIP-Q only (no unlabelled FIP was present); and 6: the negative control of 5.

In this study, we found that adding an appropriate amount of FIP-Q effectively decreased the background fluorescence, which led to the positive results being more precisely identified. Nevertheless, since FIP-Q carries quenching groups, an excessively high concentration could impede the amplification process. Therefore, optimizing the FIP-Q concentration is crucial. Building upon the optimal proportion of the composite probe FIP-FD among the total FIP primers determined above, the concentrations of FIP-Q and unlabelled FIP were further fine-tuned, as outlined in Table 6. Based on the fluorescence intensity postreaction and the initiation time, the optimal working concentrations for each subtype of FIP-Q and unlabelled FIP were determined. The results of this optimization process are visually depicted in Figure 2.

**Table 6:** Working concentrations of FIP-Q to unlabelled FIP.

Subtypes	H5 (50%)						H7 (25%)						H9 (25%)					
FIP-FD (μmol/L)	0.133						0.067						0.067					
Unlabelled FIP (μmol/L)	0.134		0.067		0		0.2		0.1		0		0.2		0.1		0	
FIP-Q	0		0.067		0.134		0		0.1		0.2		0		0.1		0.2	
Sample No. in Figure 2	+	−	+	−	+	−	+	−	+	−	+	−	+	−	+	−	+	−
	1	2	3	4	5	6	1	2	3	4	5	6	1	2	3	4	5	6

### Determining the optimal ratio of the H9 primers for the TLAMP reaction

Following the combination of the three primers, the amplification efficiency of the H9 primer set was lower than those of H5 and H7, and H9 was inhibited via multiple amplifications. Within the triple reaction system, the quantities of the H5 and H7 primer sets were held constant while the amount of the H9 primer set was optimized by adjusting the quantities of H9-FIP-Q, unlabelled H9-FIP, H9-BIP, H9-Floop, and H9-Bloop used in the reaction. This process involved increasing the outer primer to inner primer to loop primer ratio in the reaction system. The specific sequences of the H9 primer sets are outlined in Table 7. The aim of this procedure was to determine the optimal amount of the H9 primer set, ensuring that the three subtypes are amplified in a steady and independent manner in the triple reaction.

**Table 7:** Amounts of H9 primers used in the TLAMP system.

H9 primer	Concentration of H9 primers in the 25 μL system (μmol/L)					
Outer primer:inner primer:loop primer	1:8:2		1:16:4		1:24:6	
H9-FIP-FD	0.067					
Unlabelled H9-FIP	0.1		0.267		0.434	
H9-FIP-Q	0.1		0.2		0.3	
H9-BIP	0.267		0.534		0.801	
H9-F3	0.033		0.033		0.033	
H9-B3	0.033		0.033		0.033	
H9-Floop	0.066		0.132		0.198	
H9-Bloop	0.066		0.132		0.198	
Sample No. in Figure 3	+	−	+	−	+	−
	1	2	3	4	5	6

**Figure 3:**
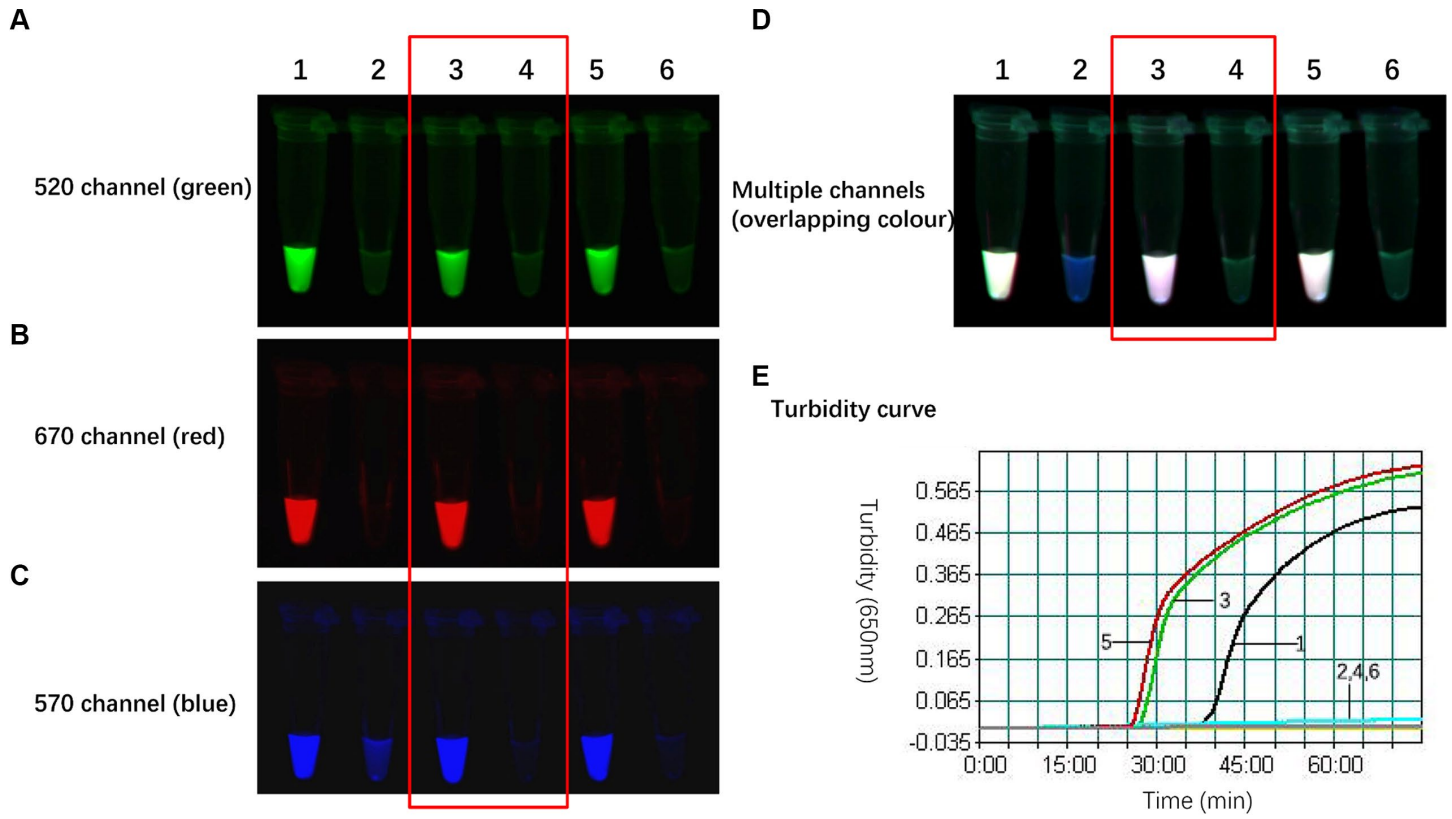
Optimal amount of the H9 primers for the TLAMP reaction. The fluorescent TLAMP products were imaged separately using the **(A)** 520 nm channel, **(B)** 670 nm channel, **(C)** 570 nm channel and **(D)** multiple channels, and **(E)** the turbidity curve was monitored with a real-time turbidimeter. 1: The ratio of H9 outer primer: inner primer: loop primer = 1:8:2; 2: the negative control of 1; 3: H9 primer set with a ratio of 1:16:4; 4: the negative control of 3; 5: H9 primer set with a ratio of 1:24:6; and 6: the negative control of 5.

As illustrated in Figure 3, the optimal working concentration of H9-FIP-FD was 0.067 μmol/L. Additionally, the optimal ratio for the H9 primer set was determined to be 1:16:4, and the initiation time was 27 min. Further increasing the amount of H9 primer did not improve H9 amplification during the reaction. After the primer combinations were optimized, each of the three subtypes could be consistently and independently amplified, demonstrating the robust performance of the TLAMP assay. After this adjustment was made, the TLAMP assay exhibited better performance and amplification.

### Determining the optimal amount of Bst for the TLAMP reaction

**Figure 4:**
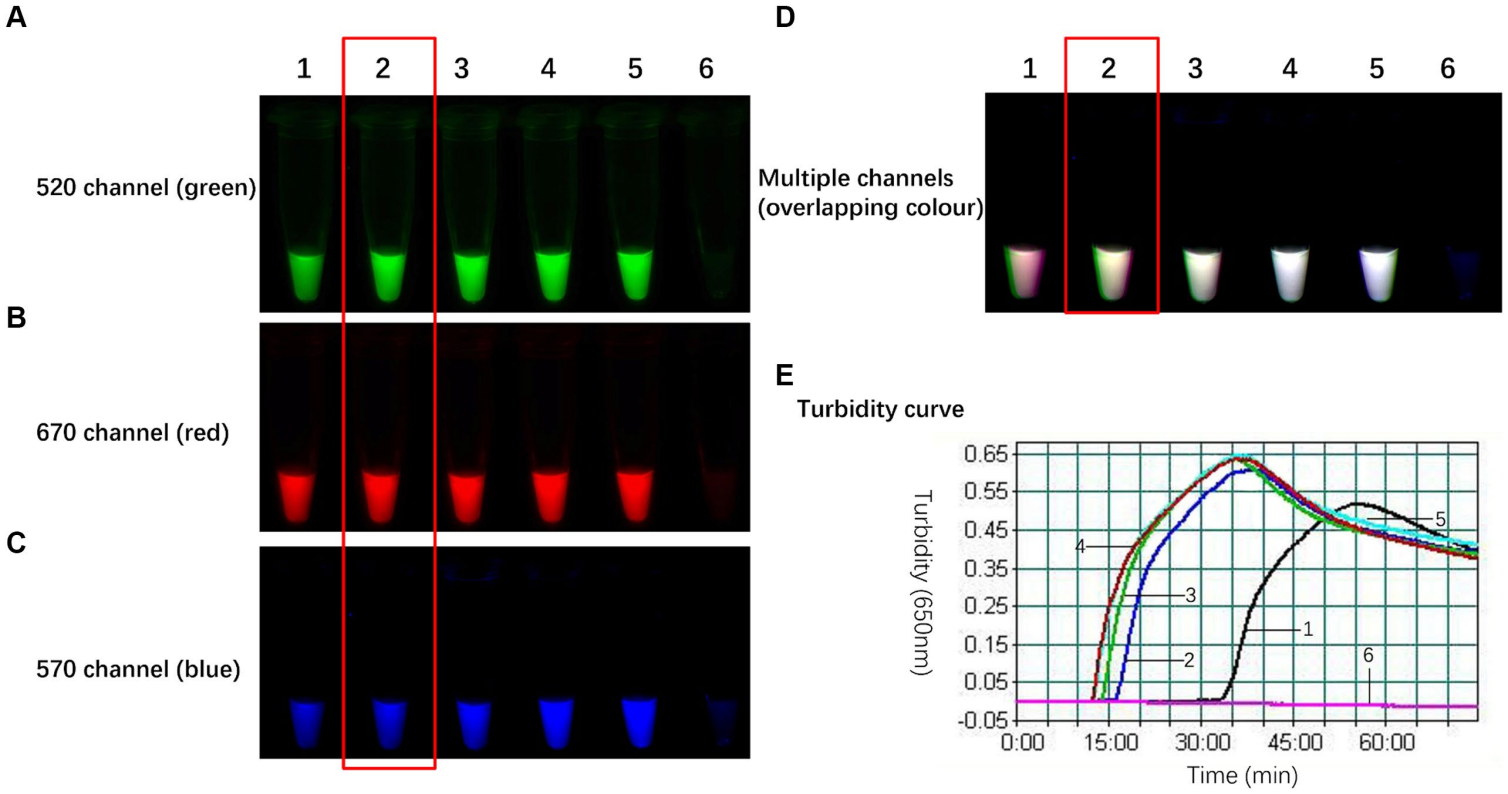
Optimal amount of Bst 2.0 WarmStart DNA polymerase in the TLAMP reaction. The fluorescent TLAMP products were imaged separately using the **(A)** 520 nm channel, **(B)** 670 nm channel, **(C)** 570 nm channel and **(D)** multiple channels, and **(E)** the turbidity curve was monitored with a real-time turbidimeter. 1: In the absence of DNA polymerase, the reaction was slow, and 33 min were needed to initiate the reaction; 2: each reaction with 2 U of Bst 2.0 had an initiation time of 16 min; 3: 4 U of Bst 2.0 was added over 14 min; 4: 6 U of Bst 2.0 was added over 12 min; 5: 8 U of Bst 2.0 was added over 12 min; and 6: negative control.

The WarmStart Multi-Purpose LAMP/RT-LAMP 2× Premix (including UDG) from New England Biolabs contains 8 mmol/L Mg^2+^, Bst 2.0 WarmStart DNA polymerase, and WarmStart RT reverse transcriptase. Although the premix performs well for single-target amplification, its efficacy is diminished during triple-target amplification due to sluggish amplification kinetics. This phenomenon arises from the coexistence of three sets of primers and probes within a single reaction tube, resulting in competitive reaction systems. Moreover, the presence of fluorophores and quenching groups inhibits amplification. Adding BST WarmStart DNA polymerase is essential for eliminating this issue because the polymerase facilitates smooth amplification of all three targets. The gradual inclusion of Bst 2.0 WarmStart DNA polymerase into the triple reaction system yielded the optimization results illustrated in Figure 4. In the absence of Bst 2.0, the TLAMP reaction exhibited slow kinetics, with an initiation time of 33 min. However, as the amount of Bst 2.0 increased, the amplification efficiency notably improved. The time needed to initiate the reaction and reach peak turbidity gradually decreased, which was accompanied by enhanced postreaction fluorescence. After 2 units of Bst 2.0 were integrated into the TLAMP system, the initiation time could be reduced to 16 min, and the postreaction fluorescence was sufficiently intense to distinguish between positive and negative samples. Further increases in the BST polymerase concentration had negligible effects on the reaction results; therefore, 2 units was established as the optimal amount of BST (2.0). This dose significantly enhanced amplification efficiency while expediting the TLAMP reaction.

### Specificity of the TLAMP assay

**Figure 5:**
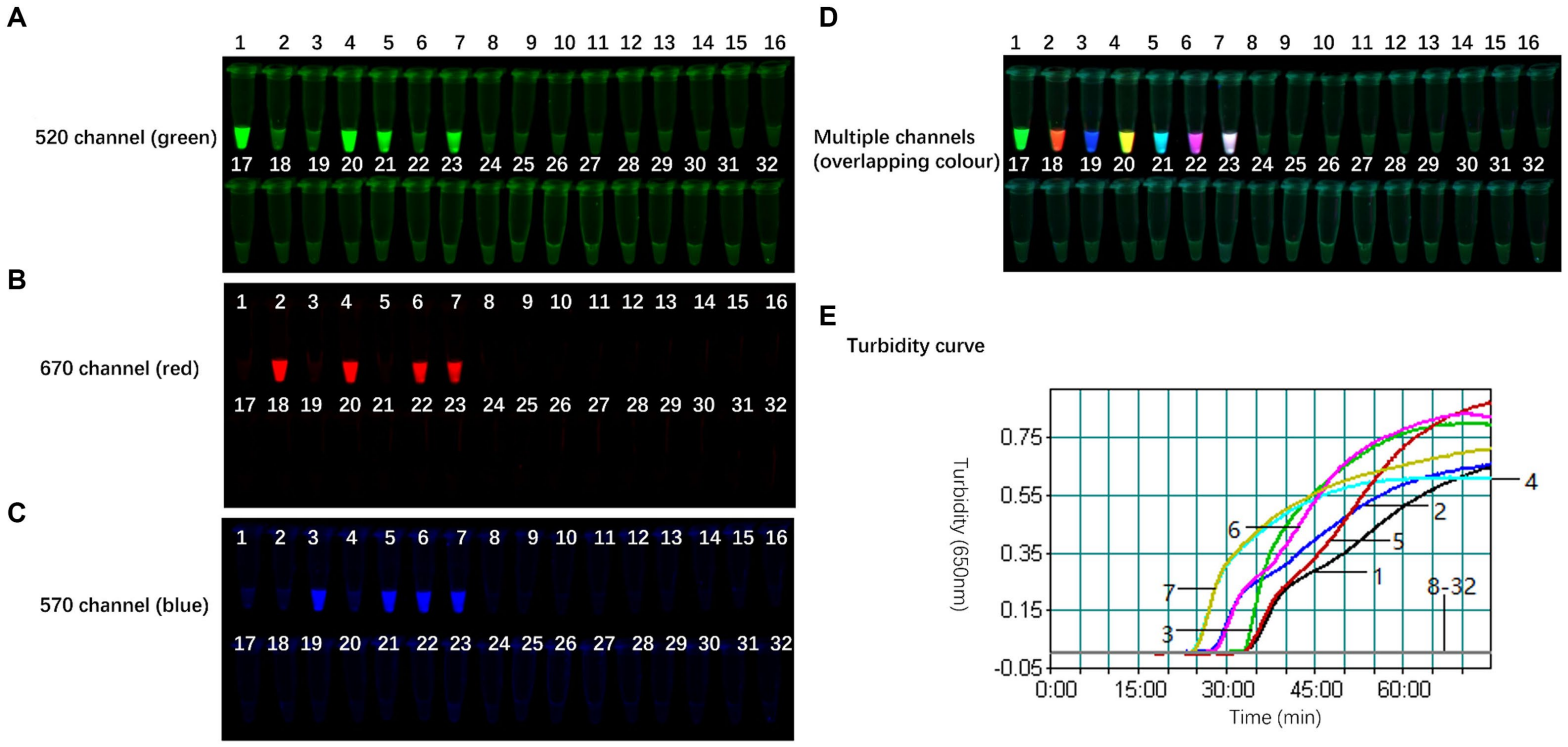
Specificity of the TLAMP assay. The fluorescent TLAMP products were imaged separately using **(A)** the 520 nm channel, **(B)** 670 nm channel, **(C)** 570 nm channel and **(D)** multiple channels, and **(E)** the turbidity curve was monitored with a real-time turbidimeter. 1: H5; 2: H7; 3: H9; 4: H5 + H7; 5: H5 + H9; 6: H7 + H9; 7: H5 + H7 + H9; 8: H1; 9: H2; 10: H3; 11: H4; 12: H6; 13: H8; 14: H10; 15: H11; 16: H12; 17: H13: 18: H14; 19: H15; 20: H16; 21: NDV; 22: IBV; 23: ARV; 24: ILTV; 25: CIAV; 26: FAdV-4; 27: ChPV; 28: MDV; and 29–32: negative control.

To assess the specificity of the TLAMP assay, AIV H1-H16 subtypes and samples artificially coinfected with H5, H7, H9, and other avian control virus nucleic acids, as listed in Table 1, were analysed using the TLAMP assay. The specificity test results are presented in Table 1 and Figure 5. H5-positive samples exhibited green fluorescence (FAM) in the 520 nm channel, H7-positive samples displayed red fluorescence (Cy5) in the 670 nm channel, and H9-positive samples showed blue fluorescence (Cy3) in the 570 nm channel. The coinfected samples exhibited overlapping colours across multiple channels. In contrast, other control viruses, including NDV, IBV, ARV, ILTV, CIAV, FAdV-4, ChPV and MDV, did not produce fluorescence or amplification across multiple channels, and the detection results were negative. These findings confirm the robust specificity of the TLAMP assay for identifying these three AIV subtypes.

### Sensitivity of the TLAMP assay

To assess the sensitivity of the TLAMP assay, eight concentrations of the RNA standard were analysed. Two microlitres of each RNA standard served as the template for testing, and 10 replicates were conducted for each standard. Table 3 summarizes the test results. Probit analysis was performed with SPSS software (SPSS, Inc., Chicago) based on the results shown in Table 3; the TLAMP detection limits for the three subtypes, along with the 95% confidence intervals (CIs), were also computed, as presented in Table 8 (27). The TLAMP assay could detect a minimum of 205 copies of H5 RNA, 360 copies of H7 RNA, and 545 copies of H9 RNA per reaction.

**Table 8:** The detection limit of the TLAMP assay determined by probit analysis.

Probit^a^	Template concentration (copies/μL), 95% CI		
	H5	H7	H9
0.95	205 (150–481)	360 (272–1,062)	545 (402–1,299)

a Proportion of replication predicted by SPSS software based on the data from the 8 RNA standards listed in Table 3 analysed with 10 replicates.

### Interference in the TLAMP assay

**Figure 6:**
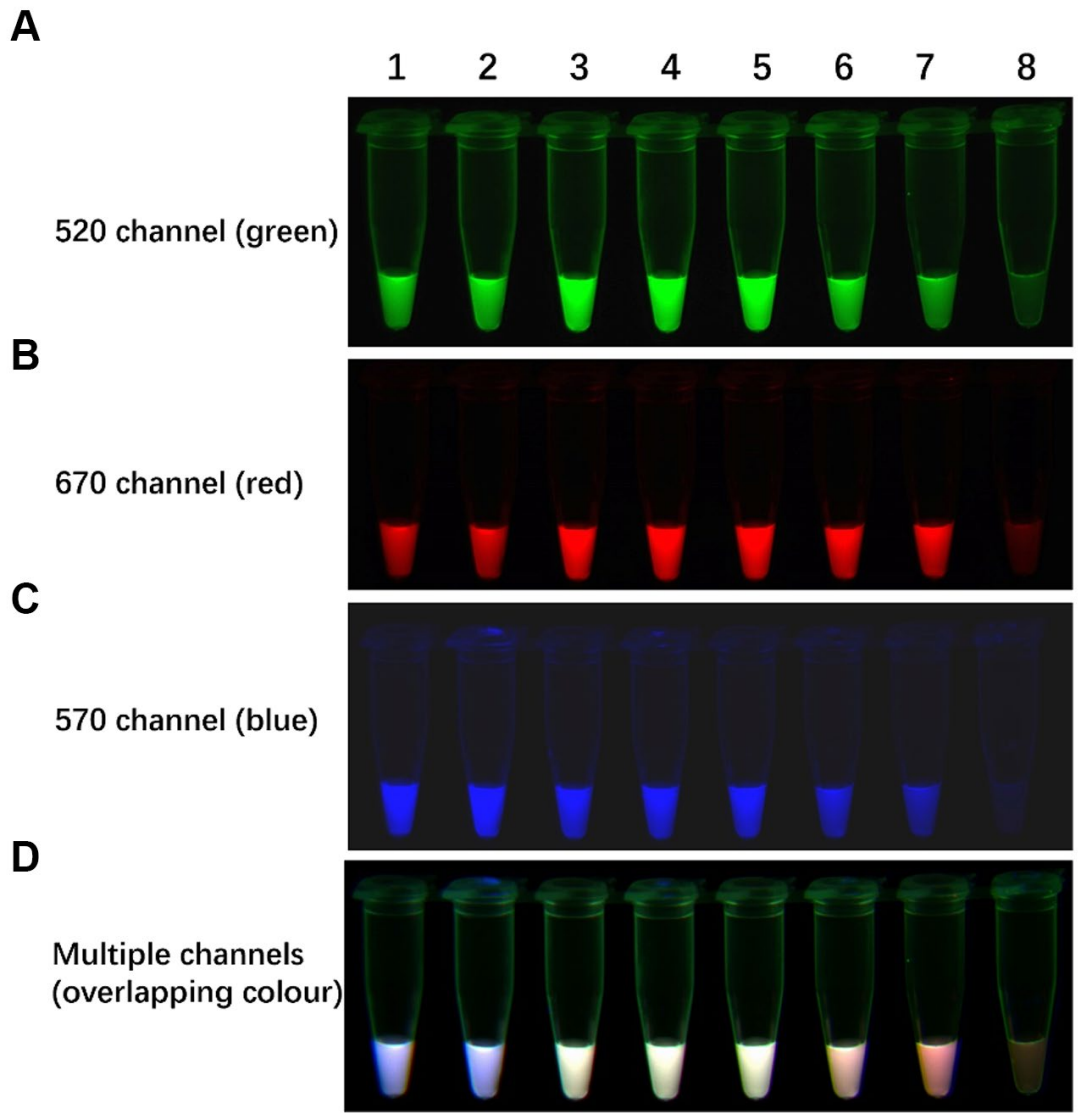
The results of the TLAMP assay. The fluorescent TLAMP products were imaged separately using the **(A)** 520 nm channel, **(B)** 670 nm channel, **(C)** 570 nm channel and **(D)** multiple channels. 1: Sample 1: H5 (10^2^ copies/μL) + H7 (10^2^ copies/μL) + H9 (10^8^ copies/μL); 2: Sample 2: H5 (10^4^ copies/μL) + H7 (10^4^ copies/μL) + H9 (10^7^ copies/μL); 3: Sample 3: H5 (10^4^ copies/μL) + H7 (10^5^ copies/μL) + H9 (10^6^ copies/μL); 4: Sample 4: H5 (10^5^ copies/μL) + H7 (10^3^ copies/μL) + H9 (10^5^ copies/μL); 5: Sample 5: H5 (10^6^ copies/μL) + H7 (10^8^ copies/μL) + H9 (10^4^ copies/μL); 6: Sample 6: H5 (10^4^ copies/μL) + H7 (10^7^ copies/μL) + H9 (10^3^ copies/μL); 7: Sample 7: H5 (10^2^ copies/μL) + H7 (10^8^ copies/μL) + H9 (10^2^ copies/μL); 8: Sample 8: negative control.

In clinical scenarios, the concentration of each subtype template in the sample under scrutiny remains unknown. Templates with higher concentrations can swiftly deplete the reaction reagents, impeding the amplification of templates present in lower concentrations. To address this issue, seven artificially coinfected samples featuring varying concentrations of H5, H7, or H9 *in vitro*-transcribed RNA were prepared and analysed using the TLAMP assay. The interference test results, as depicted in Figure 6, revealed that all the subtypes were independently amplified even though the concentrations of the H5, H7, and H9 templates differed within a single coinfection sample. After the reaction, the corresponding fluorescence was observed in the respective channel, rendering the detection results accurate and decipherable. These results underscore the minimal interference observed in the TLAMP assay, indicating that high-concentration templates within the same sample do not impede the amplification of low-concentration templates.

### Clinical application of the TLAMP assay

From 240 clinical swab samples, 26 samples were identified as only H9-positive by both the TLAMP assay and qPCR, for an infection rate of 10.8%. However, no coinfected samples were detected. The clinical results obtained both the TLAMP assay and qPCR are shown in Supplementary Table S1. Notably, the results of the TLAMP assay were 100% consistent with those of a previously published qPCR assay. The cDNA of different tissue samples from eight H5N2-infected SPF chickens donated by the University of Connecticut and the cDNA of different tissue samples from three H7N9-infected SPF chickens donated by China Agricultural University were analysed using the TLAMP assay, qPCR and sequencing, and the results indicated that the TLAMP assay could detect H5 and H7. These sequencing results corroborated the authenticity of all the positive identifications. Additionally, a cycle threshold (CT) >30 was found in 4 of the 37 positive samples. The TLAMP assay reliably detected viruses in samples amplified by qPCR at a CT <35. Notably, this TLAMP assay offers several advantages, including low cost, convenient operation, and wide applicability.

## Discussion

Due to intensive poultry farming, wild bird migration, and live poultry trading in markets, humans are in close proximity to many birds, creating favourable conditions for the increased transmission of AIVs and the emergence of new subtypes. The H5, H7, and H9 subtypes of avian influenza viruses have become endemic to domestic poultry in China and are persistent threats to public health and the poultry industry (28).

Although there have been many studies on LAMP assays for AIV detection, these methods are unable to discriminate among different subtypes. Golabi et al. (29) introduced a universal LAMP method targeting matrix gene sequences that can detect all AIV subtypes but cannot differentiate between individual subtypes. Moreover, Zhang et al. (30) developed a LAMP assay to specifically detect AIV subtypes H5 and H9 with a detection limit of 100–1,000 copies per reaction. However, this method requires fluorescence real-time PCR equipment and can only ascertain whether a sample is positive without identifying the specific subtype responsible for the positive result.

This study introduces an innovative approach involving the integration of LAMP technology with a probe. For each AIV subtype, an FD probe complementary to the F1c segment of the inner primer was designed. The FD probe incorporates distinct fluorophores, which become detached during amplification and release fluorophores with varying colours. The TLAMP results are interpreted based on both emission in a fluorescence channel and the colour and can be directly observed by the naked eye. Compared to conventional LAMP methods, the TLAMP method allows the high-throughput discrimination of multiple targets in the same reaction tube; in addition, the output is simplified and easier to interpret. The TLAMP uses fluorophores with three different emission wavelengths, presenting varied colours under different fluorescence channels. Compared to conventional methods that rely on colour change or the formation of white precipitates, the TLAMP assay produces results that can be more intuitively and accurately assessed due to the vivid contrast of the colours green, red, and blue. Furthermore, the TLAMP reaction eliminates several steps, including opening the lid, adding dye, and performing electrophoresis, which substantially reduces the risk of laboratory contamination. In future endeavours, this method could be applied in conjunction with a portable multiplex fluorescence channel analyser to develop a POC detection instrument, enabling real-time field detection at the grassroots level.

When developing a diagnostic tool, one always desires the best possible sensitivity and specificity. It is a tall order for any method, including LAMP, to outperform qPCR in this respect, since qPCR can routinely attain a sensitivity of only a few copies of the target. To date, the disadvantages of qPCR have been described in many publications, but in terms of absolute sensitivity, other methods can only approach that of qPCR. The high sensitivity of qPCR is attributed to its simple reaction system with 2 primers and one probe, complete denaturation at high temperatures, precise signal detection by a fluorescence detector, and skilled operation by laboratory personnel. However, due to the numerous primers used in LAMP assays, the samples require only a pretreatment without nucleic acid extraction, and the interpretation of the results depends on subjective judgement. Particularly in a multireaction system, competition among multiple primers and probes decreases the sensitivity, so the sensitivity of the TLAMP assay is inevitably slightly less than that of qPCR (31). In this study, TLAMP reliably detected three AIV subtypes with CT values as low as 35. Nevertheless, the reliability of CT values ranging from 35 to 38 could not be assessed in this study. Moreover, H9 sensitivity was slightly lower than that of the other two subtypes. This may have occurred because Cy3 was used as the fluorophore on H9, which exhibits lower fluorescence intensity than FAM and Cy5 (32). Consequently, in the triple reaction system, the fluorescence signal of H9 was somewhat inferior amidst the backdrop of strong fluorescence from FAM and Cy5 in multiple channels.

To successfully establish a multi-LAMP approach, the optimal quantities of primers labelled with fluorophores or quenched groups, FIP-FD and FIP-Q, must be determined. While more fluorophores lead to an increase in visual fluorescence, the background fluorescence increases concurrently, which directly impairs the interpretation of the results. Adding an appropriate amount of FIP-Q to the reaction system effectively mitigated the background fluorescence; as a result, the positive and negative reaction results were clearer, and the results could be more accurately interpreted. However, regardless of whether fluorophores or quenched groups are added to label the primers, the primers must guide the synthesis of new chains during amplification, and the labelling or quenching of primers diminishes their ability to synthesize new chains. Therefore, unlabelled FIP was added to the reaction mixture to promote the reaction. Furthermore, the components of the TLAMP reaction system are intricate, and optimizing the ratio of each primer set is critical. In conventional LAMP assays, six primers are needed to amplify a target. In the TLAMP system, amplifying three targets requires 18 primers and three probes in a single reaction system, which leads to potential competition among different subtype primer sets. If the amplification efficiency of one target primer is low, the other primers may consume the reaction components first, resulting in amplification failure. In practice, the amplification efficiency varies among primer sets. Therefore, after combining the three primer sets, it is imperative to adjust the quantity of the set with lower amplification efficiency to ensure that each target is independently and robustly amplified without influencing the other sets. In this study, we meticulously optimized the quantities of the FIP-FD, FIP-Q, and H9 primer sets and successfully identified H5, H7, and H9 subtypes of AIV.

## Conclusion

In summary, we successfully developed a TLAMP assay, which is a distinctive, sensitive, rapid, and high-throughput tool for the concurrent detection of H5, H7, and H9 subtypes of AIVs. Through this innovative approach, comprehensive prevention and control measures can be efficiently implemented, thereby reducing the incidence of avian influenza.

## Data availability statement

The original contributions presented in the study are included in the article/Supplementary material, further inquiries can be directed to the corresponding author.

## Ethics statement

The animal studies were approved by Institutional Animal Care and Use Committee (IACUC) of Guangxi Veterinary Research Institution (GVRI). The studies were conducted in accordance with the local legislation and institutional requirements. Written informed consent was obtained from the owners for the participation of their animals in this study.

## Author contributions

QF: Conceptualization, Investigation, Methodology, Project administration, Writing – original draft, Data curation, Formal analysis, Resources, Software, Validation, Visualization. ZX: Funding acquisition, Investigation, Supervision, Writing – review & editing. JZ: Methodology, Writing – review & editing. JH: Methodology, Writing – review & editing. YW: Methodology, Writing – review & editing. XL: Formal analysis, Software, Writing – review & editing. DL: Formal analysis, Software, Writing – review & editing. SL: Formal analysis, Software, Writing – review & editing. ML: Validation, Writing – review & editing. LX: Validation, Writing – review & editing. YZ: Validation, Writing – review & editing. MZ: Formal analysis, Investigation, Software, Validation, Writing – review & editing. SW: Formal analysis, Investigation, Software, Validation, Writing – review & editing. HR: Formal analysis, Investigation, Software, Validation, Writing – review & editing. LW: Formal analysis, Investigation, Software, Validation, Writing – review & editing.

## Footnotes

^1^ https://www.ncbi.nlm.nih.gov/genomes/FLU

^2^ http://primerexplorer.jp/e/
