# Supplementary material for: Simultaneous differential detection of H5, H7 and H9 subtypes of avian influenza viruses by a triplex fluorescence loop-mediated isothermal amplification assay
Source: Front Vet Sci. 2024 Jul 2;11:1419312. doi: 10.3389/fvets.2024.1419312 (PMC11250583; doi:10.3389/fvets.2024.1419312)
Supplement: Supplementary file 1 [file Table_1.DOCX]

**SUPPLEMENTARY TABLE S1 TLAMP and qPCR results for 37 positive samples**

| **Type of Chicken**  **Samples** | **Positive sample number** | **TLAMP (col or)** | **qPCR (CT value)** | **Sequencing** |
| --- | --- | --- | --- | --- |
| Tissue samples from  challenged SPF  chickens | 1 | H5+ (green) | H5+ (13.63) | H5 + |
|  | 2 | H5+ (green) | H5+ (17.51) | H5 + |
|  | 3 | H5+ (green) | H5+ (19.62) | H5+ |
|  | 4 | H5+ (green) | H5+ (18.73) | H5 + |
|  | 5 | H5+ (green) | H5+ (14.23) | H5 + |
|  | 6 | H5+ (green) | H5+ (15.03) | H5 + |
|  | 7 | H5+ (green) | H5+ (20.07) | H5 + |
|  | 8 | H5+ (green) | H5+ (22.75) | H5 + |
|  | 9 | H7+ (red) | H7+ (14.77) | H7 + |
|  | 10 | H7+ (red) | H7+ (18.26) | H7 + |
|  | 11 | H7+ (red) | H7+ (19.67) | H7 + |
| Swab samples from  LBMs | 12 | H9+ (blue) | H9+ (28.41) | H9 + |
|  | 13 | H9+ (blue) | H9+ (23.69) | H9+ |
|  | 14 | H9 (blue) | H9+ (22.72) | H9 + |
|  | 15 | H9+ (blue) | H9+ (25.82) | H9 + |
|  | 16 | H9+ (blue) | H9+ (**31.80)** | H9 + |
|  | 17 | H9+ (blue) | H9+ (21.92) | H9 + |
|  | 18 | H9+ (blue) | H9+ (29.61) | H9 + |
|  | 19 | H9+ (blue) | H9+ (28.40) | H9 + |
|  | 20 | H9+ (blue) | H9+ (**33.26)** | H9 + |
|  | 21 | H9+ (blue) | H9+ (26.85) | H9 + |
|  | 22 | H9+ (blue) | H9+ (22.72) | H9 + |
|  | 23 | H9+ (blue) | H9+ (20.55) | H9 + |
|  | 24 | H9+ (blue) | H9+ (22.57) | H9 + |
|  | 25 | H9+ (blue) | H9+ (27.31) | H9 + |
|  | 26 | H9+ (blue) | H9+ (26.42) | H9 + |
|  | 27 | H9+ (blue) | H9+ (28.56) | H9 + |
|  | 28 | H9+ (blue) | H9+ (22.77) | H9 + |
|  | 29 | H9+ (blue) | H9+ (19.36) | H9 + |
|  | 30 | H9+ (blue) | H9+ (27.17) | H9 + |
|  | 31 | H9+ (blue) | H9+ **(34.05)** | H9 + |
|  | 32 | H9+ (blue) | H9+ (19.15) | H9 + |
|  | 33 | H9+ (blue) | H9+ (28.62) | H9 + |
|  | 34 | H9+ (blue) | H9+ (15.24) | H9 + |
|  | 35 | H9+ (blue) | H9+ (23.63) | H9 + |
|  | 36 | H9+ (blue) | H9+ (26.92) | H9 + |
|  | 37 | H9+ (blue) | H9+ **(34.85)** | H9 + |
