# Supplementary material for: Simultaneous differential detection of H5, H7 and H9 subtypes of avian influenza viruses by a triplex fluorescence loop-mediated isothermal amplification assay
Source: Front Vet Sci. 2024 Jul 2;11:1419312. doi: 10.3389/fvets.2024.1419312 (PMC11250583; doi:10.3389/fvets.2024.1419312)
Supplement: Supplementary file 2 [file Image_1.pdf]

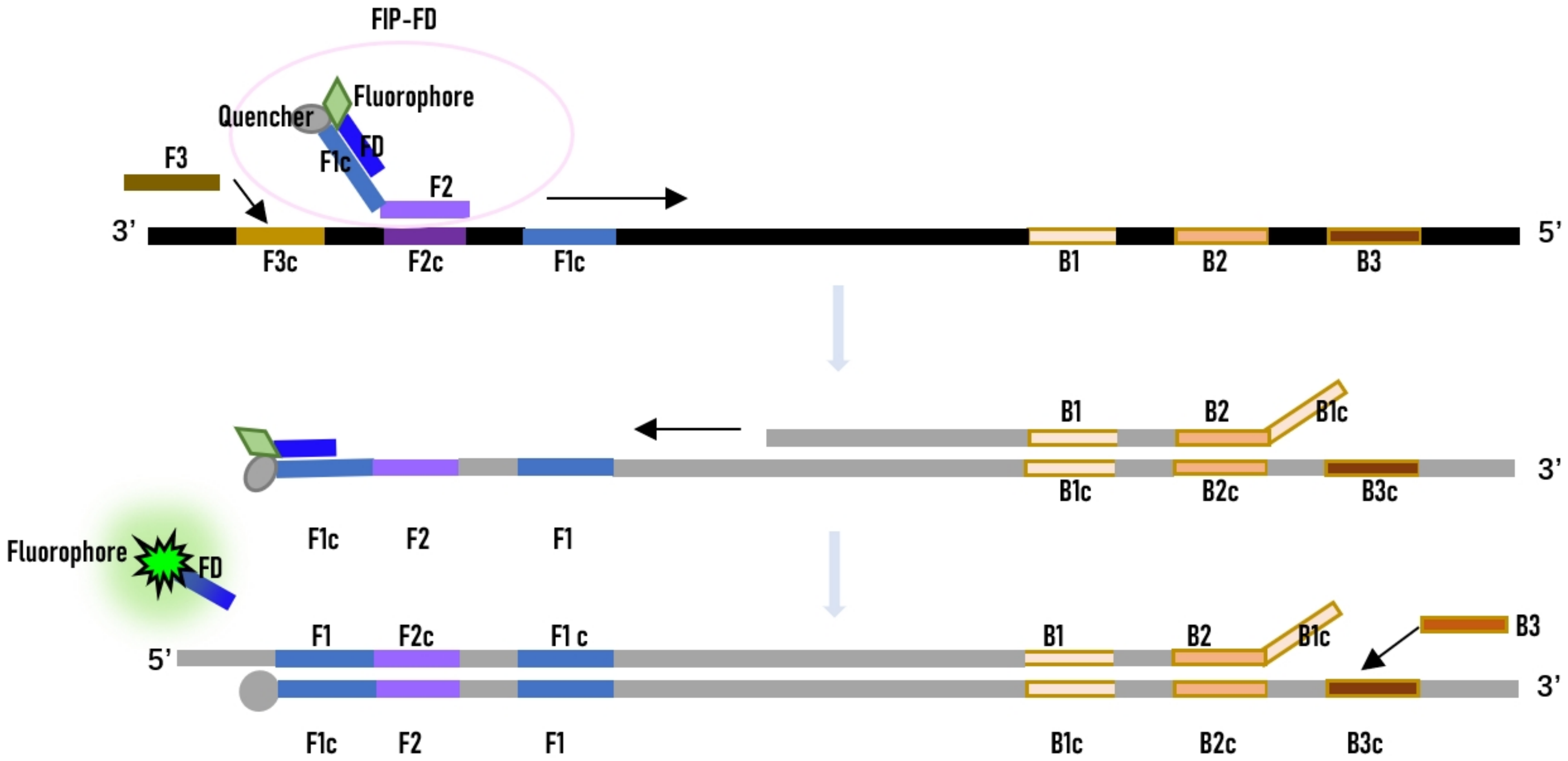

Supplementary Figure S1. Schematic diagram of the TLAMP mechanism.

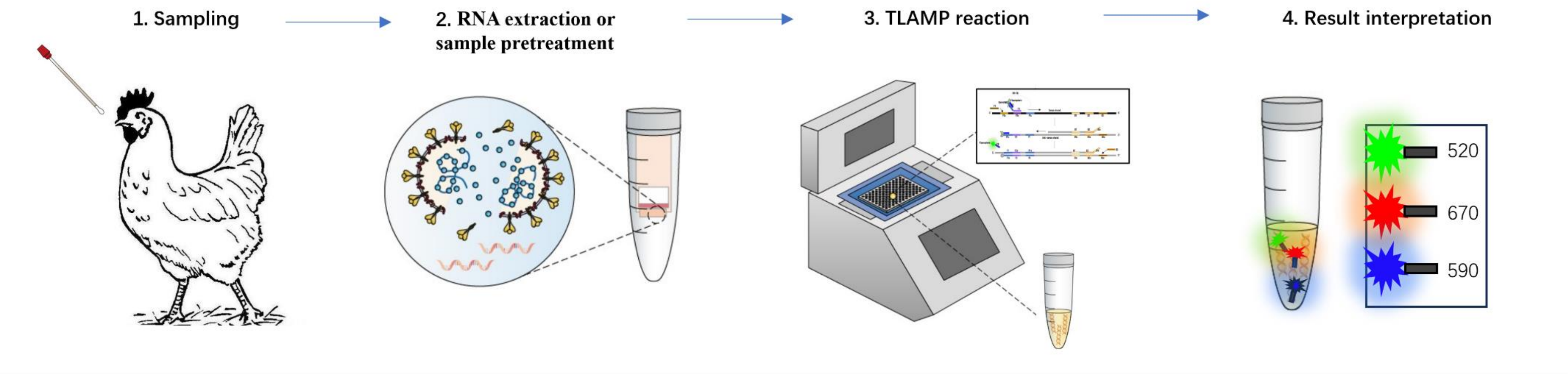

Supplementary Figure S2. Flow chart of the TLAMP assay.
